# Migrant and Refugee Youth’s Sexual and Reproductive Health and Rights: A Scoping Review to Inform Policies and Programs

**DOI:** 10.3389/ijph.2023.1605801

**Published:** 2023-06-05

**Authors:** Michaels Aibangbee, Sowbhagya Micheal, Virginia Mapedzahama, Pranee Liamputtong, Rashmi Pithavadian, Zakia Hossain, Elias Mpofu, Tinashe Dune

**Affiliations:** ^1^ School of Health Science, Western Sydney University, Penrith, NSW, Australia; ^2^ School of Medicine, Western Sydney University, Campbelltown, NSW, Australia; ^3^ Translational Health Research Institute, Diabetes Obesity and Metabolism Translational Research Unit, Western Sydney University, Penrith, NSW, Australia; ^4^ African Women Australia Inc., Liverpool, NSW, Australia; ^5^ College of Health Sciences, Vin University, Hanoi, Vietnam; ^6^ School of Health Sciences, Faculty of Medicine and Health, The University of Sydney, Camperdown, NSW, Australia; ^7^ Rehabilitation and Health Services, University of North Texas, Denton, TX, United States; ^8^ Sydney School of Health Sciences, Faculty of Medicine and Health, The University of Sydney, Sydney, NSW, Australia; ^9^ School of Human and Community Development, University of the Witwatersrand, Johannesburg, South Africa; ^10^ Discipline of Psychological Sciences, Australian College of Applied Professions, Sydney, NSW, Australia

**Keywords:** youth, human rights, reproductive health, migrant, sexual health, refugee youth

## Abstract

**Objectives:** Migrants and refugee youth (MRY) in Western nations are less likely to participate in sexual reproductive health (SRH) services. Consequently, MRY are more likely to encounter adverse SRH experiences due to limited access to and knowledge of SRH services. A scoping review was conducted to examine MRY’s understanding of and the implications for inclusive sexual and reproductive health and rights (SRHR) programs and policies.

**Methods:** A systematic search of literature across seven academic databases was conducted. Data were extracted following Partners for Dignity and Rights’ Human Rights Assessment framework and analysed using the thematic-synthesis method.

**Results:** 38 literature (peer-reviewed, 24 and grey, 14) were considered eligible for inclusion. The findings highlighted significant barriers and the under-implementation of SRHR support and services by MRY. Key policy implications include a need for programs to support MRY’s SRHR education, diversity, equity and inclusiveness and privacy protections.

**Conclusion:** The review shows that the emerging evidence on MRY SRHR suggests gaps in practices for resourcing policies and programs that promote sustainable SRH for vulnerable populations. Policies for MRY’s SRHR should prioritise programs that focus on diversity, equity and inclusion with targeted education and community resourcing strategies for sustainability.

## Introduction

Sexual and Reproductive Health and Rights (SRHR) is a fundamental human right and central to Migrant and Refugee Youths’ (MRY) knowledge and agency relating to SRHR. Migrant and Refugee Youth (MRY) refers to young individuals (between 10 and 24 years old) who have left their country of origin due to various reasons, such as conflict, persecution, or economic instability, seeking better opportunities and protection [1, 2]. International students (aged 16–24) are included as MRY because they are migrants in the country they choose to study, although their migration experience may differ [3]. The challenges faced by migrant or refugee youth, such as uncertainty regarding the length of time to settle, language barriers, cultural differences and limited knowledge and access to services in a new country, make it imperative to study the sexual and reproductive health (SRH) issues that they may face. MRY’s knowledge, experiences and agency are seldom considered [4], especially in low-resource settings [2, 5] due to their experiences of social disparities, discrimination and limited social networks and support systems in their new home country [2, 6–8]. This is despite the fact that it is clear that the pathway to optimising and improving MRY’s SRHR agency, decision-making and wellbeing outcomes can only be achieved by centring youth voices [9].

Literature shows lower levels of SRHR knowledge and literacy, limited access to social and Sexual and Reproductive Health (SRH) services, higher rates of teenage and unplanned pregnancy, and longer-lasting treatable STIs among MRY compared to their non-migrant counterparts [10, 11]. The 2018 data published by the Centre for Disease Control and Prevention [12] shows that STIs were significantly high, with an infection rate of one in five youth aged 15–24 [12]. Another 2018 study reported STI notification among youth represented 10%, 14% and 24% rise in *Chlamydia*, Gonorrhoea and Syphilis, respectively, with susceptibility higher among youth aged 15–24 (and ages 15–34 high susceptibility to syphilis), representing an estimated 80% of case occurrences despite the under-reporting of cases [13]. Within this age range, women are more likely to be diagnosed than men [13]. Although these data were not disaggregated for MRY, literature [10, 11] suggests that MRY may be disproportionately represented in these statistics. Unlike their non-migrant counterparts, MRYs are less likely to become aware of being infected, the nature and impact of STI, knowledge of and access to support services, and most likely transmit the STI inadvertently to others or suffer complications from an untreated infection as a consequence [14, 15]. This indicates significant human rights inequity, given the disparity between MRY and non-migrant youth.

### Human Rights Model and Migrant and Refugee Youth’s SRHR

The Universal Declaration of Human Rights (UDHR, 1946) outlines thirty fundamental human rights and are essential for holistic SRH and wellbeing. These rights are condensed into five principles in the Partners for Dignity and Rights (PFDAR) framework [16, 17] used to assess a program or action’s compliance with human rights principles. The principles [16, 17] are:

Universality: Affirms that quality healthcare is a fundamental human right for all (Aligns with MIPEX’s health principle) [18, 19].

Equity: Mandates equitable distribution of resources and services eliminating systemic barriers to access.

Accountability: Insists that governments establish mechanisms to enforce human rights standards, holding all entities accountable.

Transparency: Require that governments disclose all information on rights-related decisions and institutional management.

Participation: Insists on the rights of everyone to participate in decisions impacting their rights, with government support for civil society’s involvement in healthcare-related decisions.

**FIGURE 1:**
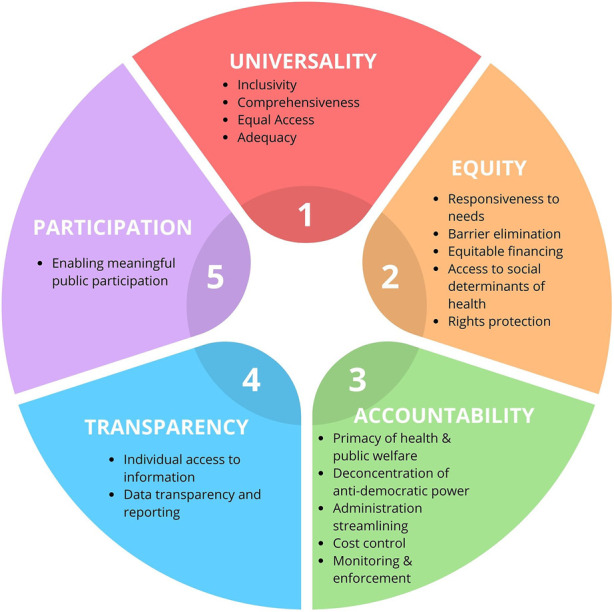
Partners for dignity and rights human rights assessment framework (Scoping Review, International, 2020–2022).

Applying the PFDAR framework to SRH underscores the need for a comprehensive consideration of human rights, including sexual rights and obligations, for wellbeing in this area. This enables the formulation of recommendations that recognise diverse identities and experiences, enhancing accessibility and efficiency of health services. It addresses specific needs and challenges faced by MRY in their SRHR context, providing better value for expenditure (Figure 1).

The PFDAR framework, with its human rights emphasis and ability to integrate the strengths of the MIPEX Health strand [18, 19] is a suitable choice for understanding and addressing MRY’s unique SRHR needs. This approach can foster a more inclusive and effective SRHR plan for MRY, ultimately promoting their wellbeing and protecting their rights.

### Research Question

This scoping review explored the emerging evidence on MRY’s sexual and reproductive behaviour and outcomes. Our specific research questions were:

1. What SRHR needs do MRY perceive in their life situations?
2. What are the policy implications of the emerging evidence on MRY-inclusive SRHR programs and practices?

Findings may clarify the role and design of MRY-focused SRHR programs that are equitably designed to support the demography.

## Methods

### Research Design

In line with Preferred Reporting Items for Scoping Reviews and Meta-Analyses extension for Scoping Review (PRISMA-ScR) guidelines [20], a systematic scoping review was conducted to identify literature (including grey and peer-reviewed literature) that investigated SRHR programs and policies for MRY.

### Search Strategy

A literature search was conducted in March 2022 across seven electronic databases, including PsycINFO, ProQuest Central, PubMed, Scopus, Web of Science and CINAHL Plus. A primary search on ProQuest Central aided in identifying keywords in titles and abstracts. Following refinement with the research team and university library staff, parameters such as location, population, and related SRH phenomena were established. For exhaustive results, supplementary searches were performed on Australia Policy Online, Google, and Google Scholar, and reference lists from selected literature were examined (Table 1). Grey literature, including reports from organizations like Family Planning and Red Umbrella Sexual Health and Human rights association, was also included to enhance the discussion [21].

**TABLE 1:** Summary of the inclusion/exclusion criteria and keywords (Scoping Review, International, 2020–2022).

Parameters	Inclusion	Exclusion	Keywords/steps
Location	Australia	Non-western countries	(Abstract) Australia OR Australasia OR Oceania OR OECD
Language	Written in English	Other languages	Select for English only
Time	Any	None	N/A
Population	Literature which includes migrant and refugee youths	Literature which focuses on mainstream populations, older people, non-refugees or non-migrant populations or Aboriginal and Torres Strait Islander	(Abstract) Refugee* OR Asylum Seeker OR Migrants OR Culturally and Linguistically Diverse OR Ethnic minority OR socially disadvantaged group OR CALD OR Cultural OR Racial OR Vulnerable group OR
			Immigrant OR Emigrant OR Boat People OR Illegal Immigrant OR Displaced Person* OR Non-Native OR Foreigner OR Foreign National OR Stranger OR Alien OR Visible minority OR Visual minority OR
			OR Non-White OR
			Expatriate OR Exile OR Newcomer OR Settler OR Escapee OR Fugitive OR Runaway OR Outcast OR Returnee OR Stateless Person
			OR Language OR English as Second Language OR Language other than English OR Language Background other than English OR English as an Additional Language or Dialect
			AND
			(Title) Youth OR Young* OR Teen* OR Adolescen* OR Young Adult
Phenomena/Target	Studies concerned with the participants’ Sexual Health, Reproductive Health and/or Rights	Not concerned with the participants’ Sexual Health, Reproductive Health and/or Rights	AND (Title)
			Sex* OR Sexually Active OR Family planning OR Sexually transmitted infection OR Sexual relationship OR Sexual prefer* OR Sexual urge OR Contracept* OR Unprotected sex OR Ovulation OR Sexual dysfunction OR Sexual Health OR Reproductive health OR Sexual Education OR Sexual health literacy OR Sexual and reproductive wellbeing OR Sexual reproductive health and wellbeing OR Sexual reproductive rights OR Safe sex OR Sexual activity OR Women and Birth OR Condom OR STI OR Prophylactic OR Pregnan* OR Unwanted Pregnancy OR Teenage Pregnancy OR Termination OR Abortion OR Long active reversible contraception OR
			Rights OR Reproductive Rights OR Sexual Rights OR Access OR Independence OR Personal choice OR Entitle* OR Prerogative OR Privilege OR Sexual Liberty OR Human rights
Study/literature type	Grey literature and Peer-reviewed academic literature		N/A
Google Modified Search			
Step 1: Refugee* OR Asylum Seeker OR Migrants OR CALD AND Youth OR Young* OR Teen* OR Adolescen* AND Sex* OR Sexually Active OR sti OR std OR sexual right AND Policy OR Policies OR Program* OR Report OR Strateg* AND Australia OR Australasia OR Oceania—first 5 pages = 50 results			
Step 2: Review full article for the above key terms with a particular focus on identifying Migrant and refugee youth specific discussion (e.g., youth, young person, adolescent, teenager) = 5 results			
Step 3: Qualitative analysis of 18 included grey literature			

The * is a function added during the search for included articles in the scientific database to include papers that has words related to the search term/s.

**FIGURE 2:**
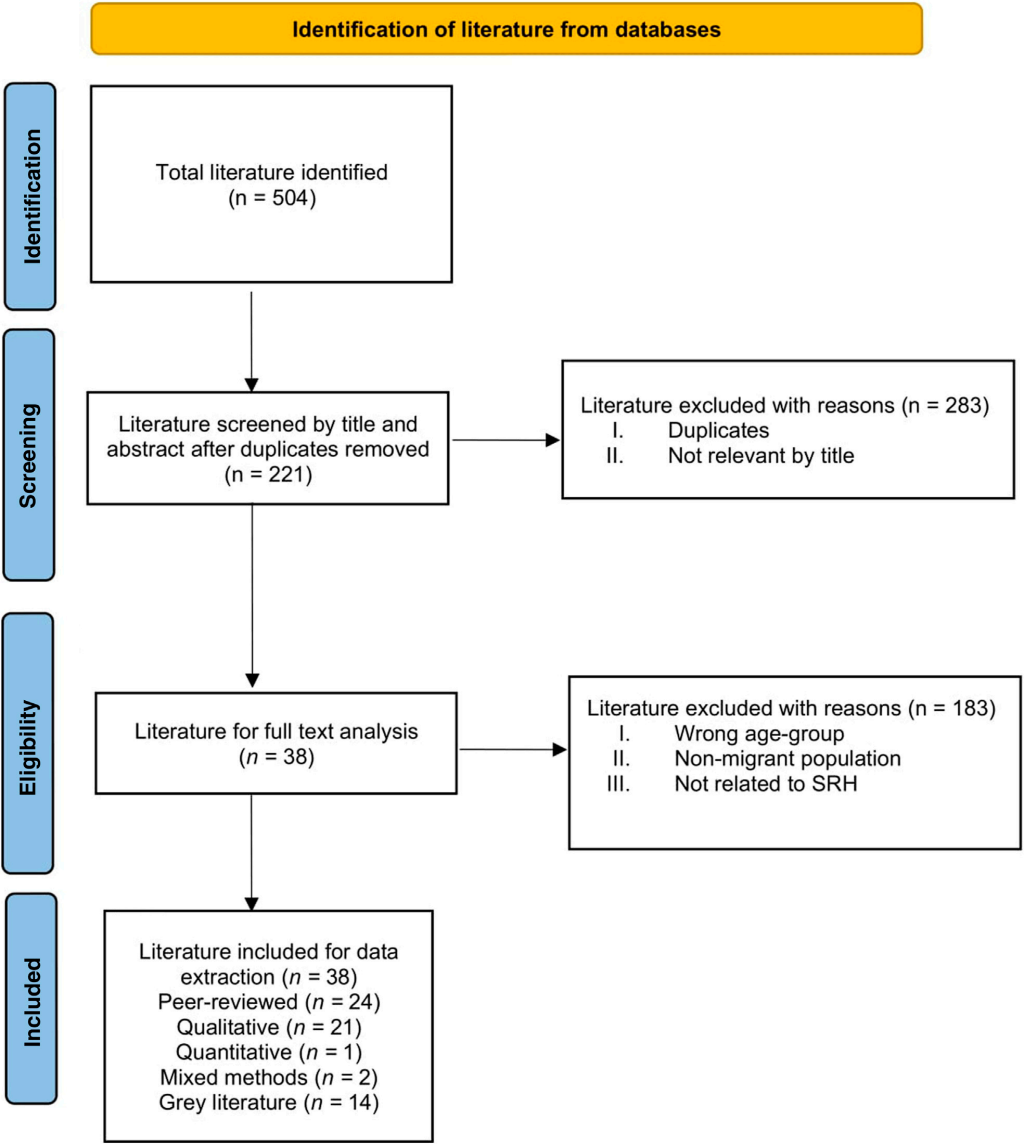
Article selection flow diagram based on the Preferred Reporting Items for Systematic Reviews and Meta-Analyses Tricco et al.’s 2018 extension for Scoping Reviews (Scoping Review, International, 2020–2022).

The search strategy details, including search terms, combinations, and the use of Boolean operators for population keywords, are summarised in Table 1. Figure 2 shows a PRISMA-ScR flow diagram of identified literature from databases.

The literature review included English-only publications without date restrictions, focusing on youth (16–24 years) of migrant or refugee background. We used SRHR-related search terms to capture both peer-reviewed and grey literature, ensuring a comprehensive and accurate review of existing evidence [22].

### Data Synthesis and Analysis

Using Thomas and Harden’s thematic synthesis method [23] (see Tables 2, 3), data from the included literature was extracted, analysed, and interpreted. This inductive process involved line by line coding of primary studies results, discussions and conclusions, developing descriptive themes, and generating analytical themes for a synthesised results presentation. All authors reviewed and agreed on the final result.

**TABLE 2:** Characteristics of peer reviewed literature included in the scoping review (Scoping Review, International, 2020–2022).

Author	Year	Study design	Setting (city not provided)	Outcome/Domain	Sample size	Age group included in review	Gender	Population background	Quality Score
Botfield et al.	2020	Qualitative	Sydney, NSW	MRY perspectives on pregnancy and abortion	27	16–24	16 females; 11 males	Multiple backgrounds (including: African, Korean, Chinese, Vietnamese)	100%
Botfield et al.	2018a	Qualitative	Sydney, NSW	MRY engagement with SRH care in General Practice (GPs)	27	16–24	16 females; 11 males	Multiple backgrounds (including: African, Korean, Chinese, Vietnamese)	100%
Botfield et al.	2018b	Qualitative	Australia, New Zealand, United Kingdom, United States	Promoting SRH among MRY using digital storytelling	28	15–24	Children, young people 18; Adult, 5; mixed, 5	Culturally diverse	100%
Botfield et al.	2018c	Qualitative	Sydney, NSW	MRY perspectives on the significance of generation on SRH care	27	16–24	16 females; 11 males	Multiple backgrounds (including: African, Korean, Chinese, Vietnamese)	100%
Botfield et al.	2018d	Qualitative	Sydney, NSW	MRY SRH information sources, and education	27	16–24	16 female, 11 male	Multiple backgrounds (including: African, Korean, Chinese, Vietnamese)	100%
					(+34 “key informants”)				
Botfield et al.	2017a	Qualitative	Sydney NSW	Engaging MRY with SRH promotion and care	23	Median: 18		Multiple backgrounds/Healthcare professionals	100%
Botfield et al.	2017b	Qualitative	NSW, VIC &	Complexities of engaging young CALD with SRH promotion	23	Median: 18	17 female, 6 male	Healthcare professionals	100%
Botfield et al.	2016	Qualitative	Australia	CALD youth and their use of services for SRH needs	120	18+		Multiple backgrounds	100%
Chung et al.	2018a	Mixed methods	Western Australia and South Australia	Young African-background women’s understandings of sexual violence and coercion	17	Median 22 years	All Female	African background (born: Zimbabwe [5], Kenya [8], Sierra Leone [2] and South Sudan [2])	60%
		[only qualitative relevant to this review]			(+81 agency participants, 23 service providers)				
Dean et al.	2017a	Quantitative	Queensland	SRH knowledge and practices among young Sudanese Queenslanders	229	16–24	80 female, 149 male	Sudanese	100%
Loftus	2008	Qualitative	Japan	Sexuality, pedagogy and gender among Japanese teen	NA	Teen	NA	Japanese	60%
Manderson	2002	Mixed methods	Queensland	Young Filipina’s SRH issues and understandings	40	14–25	All Female	Filipino	60%
McMichael	2010	Qualitative	Melbourne, VIC	Sexual health risk and protection among MRY	142	16–25	67 males, 75 females	Multiple backgrounds	80%
McMichael and Gifford	2009	Qualitative	Melbourne, VIC	Promoting Sexual literacy among MRY	142	16–25	67 Males, 75 females	Multiple backgrounds: Iraq, Afghanistan, Burma, Sudan, Liberia, and Horn of Africa countries	100%
Meldrum et al.	2014	Qualitative	Melbourne, VIC	Young Muslim women’s SRH needs and knowledge	11	18–25	All female	Mixed-backgrounds: including Saudi Arabia, Iran, Iraq, Malaysia, Fiji, Somalia, Pakistan	100%
Ngum Chi Watts et al.	2015a	Qualitative	Melbourne, VIC	Experiences of African background teen/early mothers	16	17–30 years	All Female	African background (born: Sudan [10], Liberia [3], Ethiopia, Burundi, Sierra Leone)	100%
Rawson et al.	2009	Qualitative (Grounded Theory methodology)	Australia	Influence of culture on utilisation of SRH services among Vietnamese Youth	15	18–25	All female	Vietnamese	100%
Rawson et al.	2010	Qualitative	Melbourne, VIC	Acquisition of sexual knowledge for Vietnamese-Australian Women	15	18–25	All female	Vietnamese	100%
Robards et al.	2019	Qualitative	Sydney, NSW	Healthcare equity and access for marginalised young people	41	12–24	9 female, 4 male	Multiple backgrounds: Refugees	100%
Usher	2019	Qualitative	Australia, Canada	Sexuality: MRY discourse of silence, secrecy and shame		Childhood (0–12), Adolescent [13–17]	All female	Multiple backgrounds	100%
Rawson and Liamputtong	2010	Qualitative	Melbourne, VIC	Vietnamese-Australian women’s SRH knowledge seeking, education and sources	15	18–25	All female	Vietnamese	100%
Rogers and Earnest	2014	Qualitative	Brisbane, QLD	Intergenerational experiences and knowledge of SRH among Sudanese and Eritrean women	5 young women, 8 older women, key informants)	18–30	All female	Sudanese and Eritrean	100%
Rogers and Earnest	2015	Qualitative	Brisbane, QLD	SRE (sexuality and relationships education) and SRH experiences among Sudanese and Eritrean women	5 young women, (8 older women, key informants)	18–30	All female	Sudanese and Eritrean	100%
Wray et al.	2014	Qualitative	Sydney, NSW	SRH constructions and experiences of young Muslim migrant women	10	18–25	All female	Birth country: Iraq [2], Iran [2], Afghanistan [4], Bangladesh [1] and Pakistan [1]	100%

**TABLE 3:** Characteristics of grey literature included in the scoping review (Scoping Review, International, 2020–2022).

Author	Year	Article title	Literature type (study design)	Setting (city not provided)	Outcome/domain	Sample size	Age group included in the literature	Gender	Population background	Theoretical/analytical approach
Oriti, 2017	2017	Experts say sexual slavery disturbingly common in Australia: The plight of a young African woman who sought help at a refugee centre in inner Sydney has shone a light on the problem of sexual slavery in Australia	Newspaper article	Australia	MRY (refugee) challenges (sexual slavery)	1	16–24	F	Refugee Youth (Young African Women)	N/A
U.S. Newswire, 2005	2005	American, European Youth Advocate for Reproductive Health, Rights of Young People at First-Ever US-EU Youth Advocacy Summit in Brussels	Newspaper article	United States of America	Advocacy for youth’s reproductive health rights	N/A	Not stated	Not stated	Youth (American, European)	N/A
AAP General News Wire, 2014	2014	NSW: Teen refused bail over sex assault of ex	Wire Feed (News)	Australia	MRY sexual attitude	1	16–24	M	Refugee Youth (African)	N/A
Watts, 2014	2014	Nursing: Contraception knowledge and attitudes: truths and myths among African Australian teenage mothers in Greater Melbourne, Australia	Report/Qualitative (Interview)	Australia	MRY SRH information and attitudes	16	16–24	F	African Australian Teenage Mothers	Intersectionality, cultural competency and Phenomenology/Thematic Analysis
Henderson and Conifer, 2016	2016	Refugee raped on Nauru flown to Papua New Guinea for abortion	Newspaper article	Australia	MRY experiences: sexual violence	1	16–24	F	African Refugee Youth	N/A
Kerin, 2015	2015	Iranian asylum seeker and alleged rape victim moved from Nauru to Brisbane for treatment: A 23-year-old asylum seeker	Newspaper article	Australia	MRY experiences: sexual violence	1	16–24	F	Asylum Seeker Youth (Iranian)	N/A
Walker, 2005	2005	Fed: Old male GPs making teenage contraception difficult	Newspaper article	Australia	Impact of service provider knowledge among MRY	N/A	Not stated	F	Youth	N/A
Australian Human Rights Commission, 2019	2019	Australian Human Rights Commission: U.N. Calls for National Action to Protect the Rights of Children and Young People in Australia	Newspaper article	United States	Human rights of Children and Young People	N/A	Not stated	N/A	Youth	N/A
Peta, 2015	2015	Abyan to fly back to Australia from Nauru: The young pregnant Somali refugee	Newspaper article	Australia	MRY experiences: Pregnancy and sexual violence	1	16–24	F	Refugee Youth (Somali)	N/A
Carmody, 2013	2013	Young people, sexual violence prevention and ethical bystander skills	Journal/Mixed method	Australia	Young people, sexual violence prevention	153	Median: 18	Mixed	Youth	Social Norm theory/Thematic Analysis
Nightingale, 2015	2015	Doctors call for teens to have better access to sexual healthcare: The Royal Australasian College of Physicians	Newspaper article	Australia	Service provider knowledge in healthcare	N/A	Not stated	N/A	Youth (Australian/Marginalised)	N/A
Bateson et al.	2018	Talking to young people from migrant and refugee backgrounds about sexual and reproductive health: what have we learned and where do we go from here?	Report/Qualitative (Doctoral Research)	Australia	MRY and SRH report	27	17–24	16 females; 11 males	Migrant and Refugee Youth (African, Argentine, Asian, Brazilian, Cambodian, Hazara	Not specified/Thematic Analysis
Allimant and Ostapiej-Piatkowski, 2011	2011	Supporting women from CALD backgrounds who are victims/survivors of sexual violence	Report	Australia	CALD: Sexual violence	N/A	N/A	F	CALD Women	NA
Aiyar, 2020	2020	“It’s better to have support”: Understanding wellbeing and support needs of gender and sexuality diverse migrants in Australia	Thesis/Qualitative (Semi-structured interview)	Bangladesh, Brazil, Central Europe, Iran, Malaysia, Pakistan and Philippines	MRY SRH and wellbeing	15	Median: 18	Mixed	Migrant Sexuality Diverse Youth	Intersectionality/Thematic Analysis

### Quality Assessment

The Mixed Method Appraisal Tool (MMAT) [24] was used for quality assessment of peer-reviewed literature. The studies were rated (in percentages) based on the criteria met, each worth 20% [25, 26]. The assessment process identifies potential study limitations or biases, providing a nuanced understanding of the evidence and guiding interpretation and recommendation [26]. It assures the quality and reliability of evidence synthesis on MRY’s SRHR. The assessment revealed high confidence in the scientific merits of the included literature.

## Results

Of the 504 potentially relevant articles identified, 38 literature (peer-reviewed *n =* 24, grey *n =* 14) were included in the systematic scoping review (Tables 2, 3).

### Sample

The characteristics of each literature are summarised in Tables 2, 3. Using the human rights framework [16, 17] the synthesized perspectives and reports concerning MRY cover topics like SRH education scarcity, sexual violence, limited sexual healthcare access, and social and cultural isolation. A female MRY population was the focus of 44.7% of the 17 articles, while the general MRY population comprised 55.3%. Migrant youth from thirty multicultural backgrounds and nationalities appeared in 79% of the literature, with Africa [12], Asia [8], and the Middle East [6], Europe [3] and Melanesia [1]. The review also included nine newspaper articles and three organizational internal reports, providing a well-rounded SRH perspective. These pieces shed light on government policies, SRH awareness impacts, and the social, economic, and cultural isolation of MRY. Young women’s SRHR experiences were emphasized in twelve peer-reviewed and three grey literature, tackling issues like taboos, contraceptive use, education scarcity, and sexual violence. Most of the literature (84%) was set in Australia [32], with the rest from the United States, United Kingdom, New Zealand, Canada, Asia, Brazil, and Central Europe.

### Research Foci and Theoretical Approach

Among the 38 included documents, a significant number primarily evaluated or reported on the attitudes of the community and government attitudes towards MRY’s SRH [15], SRH services [9], support to MRY’s SRHR [8] and the impact of existing policy on the MRY [9]. Other literature indirectly or directly explored the impact of existing SRH practices on healthcare outcomes [12]. Most studies implicitly or explicitly aimed to make recommendations about improving SRH support for MRY including enhancing education curriculum to include SRHR, multicultural competency training and practice, and improving SRHR awareness in practice among healthcare professionals. Only 12 documents explicitly indicated the use of a theoretical approach to guide the literature, utilising theoretical frameworks such as intersectionality, cultural competency, phenomenology, narrative, constructive grounded theory and social norm theory.

### Research Design and Methodology

Out of the 38 documents, 24 used qualitative methodology with focus groups, surveys, and interviews as common data collection strategies. Three articles [27–29] employed mixed methods, and one [30] utilised quantitative methodologies (see Tables 2, 3).

### Major Findings

Four major themes emerged from line-by-line coding of the included literature, aligning with four PFDAR [10] human rights principles (Universality, Equity, Accountability and Participation). The themes are *Limitations of Sexual Reproductive Health Education, Systemic Discrimination—Restricted Access to Sexual Healthcare, Inequity–Sexual Violence, Coercion and Exploitation, and Anonymity and Privacy Risks.* For clarity, brevity and readability, results are presented collectively with example citations for major findings.

#### Theme 1: Limitations of Sexual Reproductive Health Education

The literature highlighted limitations of SRH education as central to accessing other rights [27, 28, 31–39], impacting not only migrant youth but also the general youth population [36, 39, 40]. In line with the PFDAR human rights universality and participation measures, which advocates for unrestricted access and MRY involvement in SRH services, sub-themes like exclusionary practices within the health system [27, 32, 40] and inefficient culturally specific programs for MRY were revealed [27, 28, 36, 37, 41–43] breaching the PFDAR’s Participation principle and MIPEX’s education and anti-discrimination policies, as they require education systems to cater to the needs of migrant youth [16–19]**.**

SRH education was often found lacking in cultural understanding, negatively impacting young people’s attitudes and behaviours towards SRH programs such as family planning services and sexual health clinics. The need for culturally safe programs for MRY participation [27, 37, 43, 44] and inclusion of safe sex in national school curriculum by government and policymakers was underscored [31, 35, 45–47]. Some literature associated practices of sexual exploitation, coercion, violence and gender inequality among youth to limited SRH education and non-existent gendered policy, programs and practices [43–45, 48].

#### Theme 2: Systemic Discrimination—Restricted Access to Sexual Healthcare

Systemic discrimination [40, 49–51] manifesting as limited or delayed access to sexual healthcare and social and cultural isolation, emerged as a theme violating the PFDAR’s Equity principle. Some articles demonstrated that youth either could not access information on SRH topics or were deterred by the stigma associated with accessing sexual health services [27, 32, 34, 37, 38, 42, 45, 51–54]. Marginalized groups, like youth with disabilities and the LGBTQI+ community faced higher SRH discrimination [32, 44, 45, 51, 55]. Particularly, women experienced intersections of racism and sexism, along with the challenges of living on limited incomes, affecting service access [27].

The Accountability principle was violated, evidenced by an instance of systemic discrimination in the form of a discriminatory and inadequate police response towards a refugee youth who had been raped and was found disoriented on the street [50]. The rape survivor was held in police van for 45 min while the officers watched celebratory fireworks before taking her to the hospital. Some articles highlighted government’s negligence toward MRY rape survivors in immigration detention, resulting in higher suicide risks [15, 40, 43, 49, 50, 56].

Literature recommended acknowledging the background (life and experiences) and influences (culture, community, and family) of naturalised migrant youth (e.g., African-Australian youth) in healthcare services and programs development for MRY [28, 34, 41, 57]. This recommendation aligns with the PFDAR’s Participation principle, endorsing the inclusion of migrant youth perspectives in healthcare decision-making.

#### Theme 3: Inequity–Sexual Violence, Coercion and Exploitation

Inequity, underscored by persistent instances of sexual violence, coercion and exploitation, surfaced as a critical theme [27, 28, 33, 40, 44, 49, 50, 58]. These instances ranged from rape, forced prostitution, sexual slavery, sexual assault, victim-blaming, to trafficking and forced labour, often inhibiting disclosure due to fear of reprisal and anonymity concerns in ethnic minority communities and dissuade a reporting culture among some service providers [28, 40, 50]. Particular report highlighted international students as a vulnerable but overlooked migrant group severely affected by sexual abuse [44]. The nature of mandatory reporting further discourages disclosure among MRY due to potential legal implications and impacts on their settlement [27]. The findings also highlight gaps in government awareness of sexual slavery and forced labour, particularly in migrant and refugee communities, violating the Equity and Accountability principles of the PFDAR framework [33]. These findings illustrate the heightened vulnerability of MRY to human rights abuses due to discrimination and lack of resources, emphasising systemic barriers and inequitable access to services.

#### Theme 4: Anonymity and Privacy Risks

Findings show that cultural, community, and family taboos shape MRY’s understanding of SRH, impacting their ability to access services due to fears of judgement and breaches of anonymity, especially in closely-knit communities [32, 36, 45]. This is in variance with PFDAR’s Equity and Participation principles. Fear extends to engaging with older or culturally similar health professionals, expecting judgement or unfriendliness [32, 36, 38, 39, 45, 47].

Use of interpreters further discourages help-seeking due to concerns that interpreters could be community figures, thus violating the Equity principle. Literature emphasised youth’s rights to confidentiality in professional interactions [32, 45], aligning with the PFDAR’s Accountability principle. Other literature expressed some health professionals’ unawareness of legal age of consent may negatively impact their services, with women often facing community and practical barriers to help-seeking [27, 34, 45]. Consequently, this may deter MRY from seeking information about STI, pregnancy tests, or contraception [42, 56].

Chung et al.’s [27] focus group (*n* = 25) suggested a culturally responsive approach to reduce the stigma around discussing sexual topics openly and address violence against women [51]. Reiterating PFDAR’s Participation principle, Carmody [28] proposed using social norm theories to foster supportive normative environments to support individual’s beliefs or attitudes [41]. Further recommendations included emphasizing confidentiality through digital media, creating welcoming environment, promoting discrete waiting areas and discouraging sending confidential mail to youth’s home [36, 44], to align with PFDAR’s Equity and Accountability principles.

## Discussion

The review highlight that the challenges, interactions, and behaviours of MRY have not gained sufficient attention to support the development of culturally safe SRHR policies and programs for MRY. Results show significant misalignment with human rights principles in the framework, leading to persistent human rights challenges for MRY in Western countries [59, 60]. In relation to the Universality principle, the Australian Human Rights 2013 snapshot report [61] highlighted a significant human rights gap with respect to the treatment of refugees and people seeking asylum against Australia’s international obligation. In 2019, the Human Rights Commission [48] found that children do not have substantial rights and therefore proposed strategies to advance the rights of children [62] to align with the principle. While the action in 2019 refers to the rights of children in general, by extension, it disproportionately impact MRY due to additional obstacles such as settlement, cultural and language barriers, and education deficit. Additionally, the call for the government to discontinue the arbitrary detention of children who are refugees or seeking asylum, and invest in children’s wellbeing is also an indication of the recognisable SRH risks faced by youth [48]. Essentially, the Universality principle—which maintains human rights for, regardless of race, gender class or socio-economic status—has not been fully realised for MRY [16, 17, 63].

The findings highlight gaps in adherence to the Participation principle which ensures both civil, political, economic, social, and cultural rights. The findings indicate an apparent neglect of MRYs’ SRHR possibly due to a lack of awareness among governmental agencies (such as the police and other significant stakeholders), and fear of exploitation among victims [64]. While some literature highlighted the endemic nature of the lack of SRHR conversations, the general findings did not highlight significant advocacy or effective communication strategies at governmental or community levels respectively to highlight the plight of MRYs in addressing their SRHR concerns. Discourses identified mainly involve high-level engagements such as judicial cases or human rights conferences, with limited engagement from migrant communities, particularly the youth. This situation contravenes the Participation principle, which affirms people’s right to partake in decisions impacting them [16, 17, 65].

To uphold principles of Universality and Participation, the findings advocate for the education of key stakeholders to enhance awareness about the challenges MRY face [31, 66, 67]. Stakeholder education through organisation-wide campaigns [68] and grassroots engagement initiatives [69] is found to provide common ground for SRH conversations leading to improved programs and policies. Such engagement facilitates understanding and addressing personal biases [70] and promotes reflection on cultural sensitivity and SRH service provision [71]. Being able to access a culturally safe service can positively influence MRY’s SRH decision-making and improve help-seeking behaviour. As Dune et al. [72] suggest, effective SRH strategies should consider MRY’s experiences in a collaborative culture aimed at dismantling SRH stigma across society.

Moreover, the COVID-19 pandemic has significantly impacted MRYs’ SRHR issues, exacerbating existing barriers and inequalities in accessing SRH services and information as healthcare systems were overwhelmed [73–76]. Disruptions in education also hindered access to vital SRHR education, making it more difficult for MRY to receive accurate and culturally appropriate information on SRHR topics [74]. Additionally, the pandemic amplified the vulnerability of MRY increasing their susceptibility to sexual exploitation, violence, and other SRHR-related issues as well as heightened mental health concerns [76, 77]. Recent humanitarian crisis occasioned by the war conflicts in Europe have led to a surge in MRY populations seeking refuge in Western countries [75], further emphasizing the importance of addressing their unique SRHR needs. It is essential for researchers, policymakers, and practitioners to remain vigilant in monitoring and responding to these changing global dynamics, as they have a direct bearing on MRY’s SRHR. In light of these challenges, it is crucial to consider the MIPEX Anti-discrimination policies when discussing the PFDAR principles, in order to ensure that MRY populations are adequately supported and protected during public health emergencies and beyond.

Additional findings show a deficit in SRHR education in the education curriculum and the gravity of the impact it has on the migrant and refugee population. However, there is no evidence to show a consideration for functional SRHR education that is focused on MRY needs at a community level. The findings reveal that local organisations are working to raise awareness around STIs such as HIV, calling out risky attitudes to sexual behaviour among youth and the importance of safe sex [35, 78], aligning with studies [79, 80] highlighting youth’s minimised perception of sexual health risks and unplanned pregnancy [11, 31, 81]. Furthermore, advocating safe sex or SRH-related practices is thought to be significantly confronting for faith-based institutions, although, religious institutions and communities are best positioned to influence health promotion among adherents [82]. While considering the challenges for faith-based institutions to contemplate a healthy approach to SRH, the same can be said of ethnic minority families, religious communities, and some older generation health professionals with a cultural cringe about contraception [34], thus making access to contraception difficult for youth. Thus, conscious SRH risk acknowledgment and advocacy among these communities concerning MRY is paramount.

In addition to the lack of SRHR awareness, the issue of sexual violence, coercion, and other sex-related practices are seldom discussed because of the complex implications for victims and their families [27, 83]. In tandem with included literature [83, 84]^,^ Keygnaert et al.’s [85] work revealed that sexual violence and coercion continue to impact migrant women substantially extending to psychosocial harm such as shame, vilification, community and practical barriers to help-seeking, and mental health complications including anxiety, depression, poor self-image and suicidal ideation [86–88]. This psychosocial impact infringes upon the participation and universality principles, emphasising that lack of SRHR awareness drives vulnerability to sexual vices, hence the need for improved SRHR awareness to prevent such vulnerabilities within migrant and refugee communities, including LGBTQI [33, 44, 84]. Sexual violence risks also apply to international students, a majority of whom are women, who are seldom included in the conversation [88, 89]. The findings which are also supported by Wallace’s [90] work, show that the awareness of sexual slavery in Western industrialized nations is very low despite a high prevalence of such crimes [91, 92]. Therefore, the integration of MIPEX Anti-discrimination policies [18, 19] with the PFDAR principles [16, 17] is necessary to provide support and protection to, thereby reducing their vulnerability to sexual exploitation and violence.

Therefore, addressing the SRHR issues among MRY requires a holistic approach that involves not only policy changes, but also cultural shifts, community engagement, and education. By acknowledging the unique challenges faced by MRY and incorporating PFDAR human rights principles in conjunction with the MIPEX policies, it is possible to create a more inclusive and equitable society where the rights and wellbeing of all individuals, including MRY, are respected and upheld.

### Implications for MRY SRHR Policies, Programs and Practices

SRH education curriculum: Findings suggest that incorporating comprehensive SRH education into the national curriculum is crucial for fostering SRH discussions in education and enhancing SRH knowledge and service use among young people, including MRY [93, 94]. Government and policymakers are encouraged to prioritise SRH education, which could inspire service uptake and further research opportunities for improved policy and practice [93, 94].

Workforce training: Service providers can benefit from training to encourage reflective practices that address potential biases or knowledge gaps [95, 96]. Increased cultural representation among educators and service providers, as suggested by Galagan [97] and Mitchell et al. [98] can enhance service quality and uptake.

Co-design and peer-to-peer education: Involving MRY in shaping SRHR policies and programs is potentially beneficial in empowering them, ensuring culturally responsive solutions [85, 86]. Also, peer education can effectively raise SRH awareness and promote healthy behaviours among MRY [99, 100].

Government commitment to children’s human rights obligations: The review shows significant policy gaps affecting young people’s rights, especially MRY populations. This calls for a governmental review of SRH policy to align with human rights and MIPEX anti-discrimination policies. The aim is to encourage youth participation, including MRY, in SRH decisions. Current child protection and mandatory reporting policies do not adequately consider cultural needs, causing privacy concerns and potential vilification. Parton et al. [101] referring to the child protection policy in England which has experienced high-level criticism, deemed it “a moral panic if not a witch-hunt,” [101] while Redleaf [102] holds that it traumatises families. Therefore, a co-design policy approach with migrant communities is essential [99]. Such a policy revamp would boost MRY’s confidence in understanding their rights, reporting SRH crimes, and enhance their trust in lawmakers and government agencies.

### Limitations of the Study and Future Directions

The study provides valuable insights into SRH support needs and policies for MRY but has several limitations. Despite its comprehensive blend of peer-reviewed and grey literature, the review does not sufficiently incorporate MRY perspectives or solutions based on their experiences. This gap undermines the potential of policies and services to fully address their needs. The review overlooks certain underrepresented groups within MRY, such as those with physical disabilities or neuro-divergence. This lack of representation could result in biased policy recommendations that do not cater to the diverse needs within the MRY population.

A significant limitation is the lack of intersectional analysis that considers factors like gender, sexual orientation, ethnicity, and socio-economic status. Without such analysis, it is challenging to develop comprehensive SRHR policies that address the unique challenges MRY face due to the interplay of these factors. The literature reviewed also shows a disproportionate representation of Australian MRY SRH literature, which reflects a specific sociocultural, political, and economic context even though multiculturalism has not been fully represented given Australia’s short, yet growing migration history for the majority of cultural communities. This implies that MRY’s SRH experiences and views in other industrialised countries with varying degrees of histories and experiences of migration are not fully represented in the findings against the Australian context. This over-representation could potentially limit the breadth of understanding and applicability of findings to other cultural contexts or countries with different migration histories.

The potential role of technology and digital media in addressing MRY’s SRHR needs, particularly in the context of COVID-19, is not highlighted. As many young people have access to smartphones and the internet, technology could offer new opportunities for information dissemination, support, and service provision.

Lastly, bias exists where the studies were limited to those only published in English (when trying to make inferences about MRY, or giving consideration to MRY groups in non-English speaking countries such as France, Greece, Spain, Italy, to list a few, due to the large number of migrants they receive) potentially skewing perspectives and limiting a truly global understanding of MRY’s SRH experiences. This is particularly significant as MRY groups often come from non-English speaking backgrounds.

Future research should address these gaps, incorporate a more intersectional approach, engage directly with MRY, consider the role of technology, and broaden linguistic and geographical scope to provide a more comprehensive understanding of MRY’s SRH experiences and needs.

### Conclusion

Promoting increased health literacy, safer sex practices, and a strong awareness of MRY rights, requires a comprehensive acceptance and integration of diversity across systems, institutions, and practices. An important consideration involves disrupting the intergenerational perpetuation of ineffective, often western-centric top-down SRH systems in migrants and refugees’ communities. Within the scope of this study, such disruption is achievable by prioritising youth perspectives and collaboratively designing policies and programs that adequately address their SRHR needs and rights. Essentially, healthcare systems, policymakers, and practitioners should adopt culturally competent principles that cater to diverse needs and forge effective alliances [42, 103]. This commitment is key in supporting MRY’s health and wellbeing [104] and fostering global health equity and social justice. Therefore, collaborative efforts from international stakeholders are necessary to develop culturally appropriate and responsive policies addressing MRY’s unique needs globally.

Furthermore, acknowledging the limitations of the existing literature (including the need for intersectional analysis, diverse perspectives, and consideration of technology’s role in SRHR) particularly during the COVID-19 pandemic) is critical. Addressing these limitations can foster effective global policies and improve MRY’s health and wellbeing. Hence, an inclusive approach prioritizing youth perspectives, cultural diversity, and global context is required to effectively address MRY’s SRHR needs.
